# Beyond Broadway: Analysis of Qualitative Characteristics of and Individual Responses to Creatively Able, a Music and Movement Intervention for Children with Autism

**DOI:** 10.3390/ijerph16081377

**Published:** 2019-04-17

**Authors:** Kimberley D. Lakes, Ross Neville, Spyridoula Vazou, Sabrina E. B. Schuck, Katherine Stavropoulos, Kavita Krishnan, Irene Gonzalez, Kayla Guzman, Arya Tavakoulnia, Annamarie Stehli, Andrew Palermo

**Affiliations:** 1 Department of Psychiatry & Neuroscience, University of California, Riverside, CA 92521, USA; 2 School of Public Health, Physiotherapy and Sports Science, University College Dublin, Dublin 4, Ireland; ross.neville@ucd.ie; 3 Department of Kinesiology, Iowa State University, Ames, IA 50011, USA; svazou@iastate.edu; 4 Department of Pediatrics, University of California, Irvine, CA 92617, USA; Sabrina@uci.edu (S.E.B.S.); kkrishn1@uci.edu (K.K.); ireneg1@uci.edu (I.G.); kmguzma1@uci.edu (K.G.); atavakou@uci.edu (A.T.); astehli@uci.edu (A.S.); 5 Graduate School of Education, University of California, Riverside, CA 92521, USA; Katherine.Stavropoulos@ucr.edu; 6 Clare Trevor School of Arts, University of California, Irvine, CA 92617, USA; palermoa@uci.edu

**Keywords:** autism, ASD, autism spectrum disorder, music, movement, physical activity, intervention, self-regulation, cognition, executive functions

## Abstract

Movement in response to music represents one of the natural social environments in which physical activity occurs. The study of music and movement, including dance, requires a careful, holistic consideration of many features, which may include music, physical activity, motor learning, social engagement, emotion, and creativity. The overarching goal of this manuscript is to examine qualitative characteristics of and individual responses to a music and movement intervention (Creatively Able) for children with Autism Spectrum Disorder (ASD). We provide a description of Creatively Able, illustrating how the program design and physical and social environment were informed by children’s needs and preferences in order to provide an enriched environment in which to promote multiple systems in children with ASD. Using data from two pilot studies with 20 children with ASD, we illustrate how researchers can use observational research methods to measure important aspects of the social environment (e.g., children’s engagement during intervention sessions) as well as engagement of potential underlying behavioral mechanisms (e.g., self-regulation) that might reduce clinical symptoms. We further illustrate how individual responses to intervention (e.g., improvements in behaviors or symptoms) can be studied in physically active interventions. Our pilot study results showed group-level reductions in Stereotyped and Compulsive behaviors of 8% and 4%, respectively; posthoc analysis revealed that there were substantial individual differences in children’s responses to the intervention. This research illustrates robust methods that can be applied to intervention research to improve our understanding of important features of interventions that might help promote development in various domains, including executive functions and self-regulation.

## 1. Introduction

Autism Spectrum Disorder (ASD) is a neurodevelopmental disorder characterized by persistent deficits in social communication and interaction and restricted, repetitive patterns of behavior, interests, or activities that are present during a child’s development and cause clinically significant impairments in functioning [1]. The prevalence of ASD in children in the United States is estimated to be 1.47% [2], and interventions to improve functioning in children with ASD are an important research priority. In this manuscript, we examine the potential of Creatively Able, a music and movement intervention, to promote development in children with ASD. The theoretical rationale for Creatively Able integrates science from multiple disciplines, including physical activity and exercise science, music and the arts, clinical psychology, and developmental psychology. 

### 1.1. Physical Activity in Children with Autism

It is widely understood that physical activity (PA) is important for child development, yet levels of PA are insufficient for many groups of children [3], particularly children with disabilities who engage in less PA than their typically developing peers [4,5]. There is evidence that children with ASD engage in less PA than their peers [5], and comparisons between children with and without ASD indicate that children with ASD spend more time engaged in sedentary behaviors, which is linked to greater use of screen time and Body Mass Index (BMI) [6]. Children with ASD who do not engage in PA regularly are more likely to have an unhealthy weight [7] and to be less physically fit [8]. Among children with ASD, older children engage in less PA than younger children [9], suggesting that the gap may widen as children grow older. A majority of parents of children with ASD (74%) seek complementary and alternative medicine (CAM) treatment approaches [10], including PA interventions [11], often motivated by concerns with the safety and side effects of prescribed medications [10]. However, to date, there are few studies evaluating the outcomes of PA interventions for children with ASD, making the development and study of intervention programs that promote PA for children with ASD an important research priority.

### 1.2. Physical Activity or Movement Interventions: Considering Contextual and Qualitative Factors

There has been an increase in the number of PA or movement interventions in youth over the last 20 years, with a focus on the impact of PA programs on important developmental outcomes, including executive functions [12,13]. However, very recently, there has been a growing recognition that in order to understand how PA programs impact executive functions, it is necessary to move from merely focusing on exercise dose or intensity toward the examination of qualitative elements of PA, such as cognitive, motor coordination, and complexity demands that could potentially contribute to the impact of PA interventions on executive functions [14,15,16,17,18]. A recent review and meta-analysis attempted to provide insight into the impact of different characteristics of PA by categorizing PA interventions into groups based on primary features of the interventions (i.e., aerobic, motor skills, cognitively engaging activities, and all combinations of those facets) [19]. Results from the meta-analysis highlighted the significant positive effect of PA interventions on executive functions (0.46 effect size) and identified differences between the categorically different PA interventions compared to various control groups (e.g., no treatment, academic content, physical education). However, high heterogeneity among studies provided support for what other researchers have suggested—there are a large number of factors that can contribute to the impact of PA interventions on executive functions, including unique elements within a PA program (e.g., rhythmic movement, mental strategies, emotional activation) as well as contextual factors, such as the physical and the social environment. In a review of interventions to promote executive functions (including, but not limited to, physical activity interventions), Diamond [20] encouraged researchers to examine the hypothesis that the most effective interventions will be those that, “(a) train and challenge diverse motor and executive function skills, (b) bring joy, pride, and self-confidence, and (c) provide a sense of social belonging (e.g., group membership) (p. 963).”

PA programs that include music challenge diverse motor and cognitive skills as participants need to mentally work with the tempo, learn new rhythmic movements and motor sequences, and stay in tune with the music. Music and rhythmic programs have been found to be important for speech and are suggested to benefit literacy skills in addition to improvements in PA levels, especially in the early years of a child’s development [21,22]. While PA programs with rhythm and music are understudied in the literature, they are hypothesized to improve executive function skills [23]. Thus, we anticipated that Creatively Able might have the potential to positively affect development in children with ASD in numerous domains.

### 1.3. Music Interventions for Children with Autism

The growing interest in music interventions for children with ASD has been in part inspired by research hinting that music might be a potential vehicle for emotional understanding [24,25] and emotion regulation [26,27] in ASD. There is growing evidence that music interventions can improve ASD symptoms and comorbidities in children with ASD [28] as well as social interactions, communication, and emotional engagement [29,30,31]. Given these findings, it is unsurprising that the interest in music as a therapy for ASD has gained traction in recent years, and that children with ASD have been identified as a potential clinical group that might benefit from music-based interventions [31]. We do not yet know if music and movement interventions provide additional benefits over music interventions without movement, and our research aims to begin to address these gaps in research, by examining an intervention that integrates music with physical activity or movement. 

### 1.4. Creatively Able: A Music and Movement Intervention for Children with ASD

Creatively Able is a novel approach to engaging children with ASD in the musical arts, which was developed by Andrew Palermo, a Broadway artist and professor. Creatively Able was designed to engage children with ASD by focusing on their strengths and interests. Music is carefully selected for the intervention based on key criteria (e.g., having an easy-to-follow 4-count rhythm) that enable the instructor to eliminate potential distractions and provide a simple structure that facilitates teaching children rhythm and expressive story telling through music and movement. Selected songs (e.g., Wild Horses by Bishop Briggs) have a steady rhythm, and the melodic structure and overall production elements are not so powerful as to over-stimulate hypersensitivity, which is common in children with ASD. Oftentimes, music is used without lyrics to reduce the potential distraction of lyrics. Each session begins with activities that include a warm-up with across-the-floor movements and a mirroring activity where children are paired with one another and learn to move in sync with one another. The primary activity in each session involves identifying a theme derived from participant interests (e.g., dinosaurs) and developing a story that is then physically expressed to music selected by the instructor. Each participant creates 8 counts of the musical performance (creating movements to continue the story from where the prior 8 counts left off). The final result is a story physically expressed to music. The group performs and practices the piece until the end of the session, which we hypothesize promotes executive functioning (e.g., selective attention, working memory) as children need to attend to, remember, and subsequently perform each other’s segments of the performance piece.

The intervention was uniquely designed to introduce children to music and engage them in physical activity in a social environment that promotes creative expression and social communication, a key deficit in individuals with ASD. For example, in the “mirroring” activity, children with ASD are paired; they each have a turn to be the “leader” and the “follower”. The leader will move his body in any way he chooses; the follower stands facing the leader and mimics his movements. This continues for several minutes until they switch roles. Interestingly, participants make eye contact and closely follow one another; the Social Motivation hypothesis suggests that children with ASD lack motivation to engage in social activities (e.g., joint attention, making eye contact) because they find these activities less rewarding when compared to their typically developing peers [32]. Creatively Able aims to provide the motivation for children to engage in social activities that promote joint attention, eye contact, and awareness of others and gives them opportunities to practice these skills in each session. Thus, Creatively Able is innovative in how it applies music and movement to address deficits in children with ASD. 

The overarching hypothesis driving our research is that music and movement interventions provide a multisystem learning opportunity characterized by embodied cognition, which should enhance cognitive outcomes, including executive functions. The embodied cognition view holds that cognitive processes are deeply embedded within the ways in which our body interacts within the world [33]. In this context, expressive movement is likely to accentuate the beneficial effects of both music (because the child is moving with the music as opposed to simply listening) and physical activity (as moving to music rather than simply moving might engage additional cognitive processes). Moreover, we expect that other features of the intervention—motor learning and social activities—will have a positive effect on outcomes. Our target outcomes include ASD symptoms (e.g., stereotyped and compulsive behaviors), self-regulation, and executive functioning. Importantly, repetitive or stereotyped behaviors are one of two primary symptoms of autism. These symptoms, traditionally known as restrictive and repetitive behaviors (RRBs) have been related to deficits in executive function [34,35,36,37]. Executive function is a broad term for higher-order processes including inhibitory control, attentional flexibility, response monitoring, and working memory (see Diamond, 2013 for a review [38]). Response monitoring is critical to the ability to evaluate one’s performance and make appropriate adjustments when necessary for a desired outcome. Difficulties monitoring one’s own performance, or insensitivity to response outcomes, help explain repetitive behaviors in ASD, as well as difficulties with flexibility and self-regulation. While music and movement interventions may be important for a variety of physical, psychological, and developmental outcomes, we narrowed our focus for the current pilot study. In this research, we predicted that Creatively Able, a music and movement intervention for children with ASD, would improve ASD symptoms (e.g., stereotyped and compulsive behaviors). Moreover, in this pilot research, we also predicted that children would respond positively to the intervention, as evidenced by self-reported ratings of their enjoyment during intervention sessions. Finally, we predicted that the intervention would engage targeted outcomes, as evidenced by research observer ratings of children’s engagement and self-regulation during intervention sessions.

## 2. Materials and Methods

### 2.1. Recruitment and Participants

This study was approved by the Institutional Review Board of the University of California, Irvine (UC Reliance #3111). Written informed consent was obtained from parents, and children provided verbal assent. Boys and girls ages 7 to 12 years with a history of a diagnosis of ASD were eligible to participate. Inclusion criteria included: Ability and willingness to participate in group music and movement classes, having a parent able to consent in English, and child’s ability to assent in English. Exclusion criteria included: Severe visual impairment, severe physical disabilities, or medical diagnoses that would preclude participation in the intervention. 

Study 1 included 12 children (42% female); all children had prior diagnoses (provided by community providers) of ASD, and 44% were also diagnosed with Attention Deficit/Hyperactivity Disorder (ADHD). The Study 2 sample (*n* = 8) included 4 females and 4 males with ASD, with some participants having comorbid disorders (28.5% ADHD, 14.3% Anxiety Disorders, and 14.3% a Long Term Memory Disorder). Diagnoses were reported by parents at enrollment and had been obtained through community providers as part of qualification for services at the center where recruitment was conducted. The mean participant age was 8.5 ± 2.07 years.

### 2.2. Creatively Able Pilot Intervention

In Study 1, we offered two sessions over two consecutive Saturdays, aiming to first test the feasibility and acceptability of the intervention before conducting a pilot study. In Study 2, a 4-week pilot intervention for Creatively Able was delivered; participants attended eight forty-five minute sessions. Sessions were offered two times per week during after-school hours and were held in a university dance studio. Professor Palermo and a female assistant taught all sessions. Parents waited outside the studio for the duration of the classes.

### 2.3. Assessment of Intervention Sessions: Qualitative Features and Individual Engagement

To evaluate whether or not the intervention engaged behavioral targets that we predict might be underlying mechanisms of change in ASD symptoms (e.g., self-regulation of emotion), we evaluated video-recorded sessions; each session was rated across three parts reflecting shifts in group activities: Warm-up/Across the Floor, Mirroring, and Creative Choreography (e.g., developing a story that is physically expressed to music). The first, last, and middle sessions were individually rated for student engagement [39] based on the following criteria: Verbal participation, positive display of emotion, active effort in the class, focused attention, and persistence. Students were also rated using the Response to Challenge Scale (RCS) [40,41], an observer-rated measure of child self-regulation. Both scales are 7-point rating scales, with possible scores ranging from 1 to 7; the midpoint of the scale (4) represents “average” for a typically developing child of that developmental age. Eleven trained research assistants viewed the video recordings and rated each child during each session. We aggregated scores across raters (i.e., averaging 11 raters’ ratings for each item to yield one score per item per child); analyses of scale data demonstrated high internal consistency and strong rater agreement. 

### 2.4. Assessment of Individual Outcomes

Participants in Study 1 were asked to commit 4 h (2 visits) to this study: Baseline assessment and consent/assent (1 h) followed by one session and a second assessment and session one week later (1 h). Participants in Study 2 were asked to commit 10 h (10 visits) over a period of five to six weeks to the study: Baseline assessment and consent/assent (1.5 h), eight intervention sessions over four weeks (45 min sessions two times per week), and one post-intervention assessment (1 h).

A demographic questionnaire was used at enrollment to gather information on the child’s diagnoses, racial/ethnic background, primary language spoken in the home, parent educational level, and family income. 

Parent reports of children’s ASD symptoms were measured using the Repetitive Behavior Scale-Revised (RBS-R) [42]. The RBS-R is a 43-item questionnaire designed to assess six categories of behavior, including stereotyped behavior, self-injurious behavior, compulsive behavior, ritualistic behavior, insistence on sameness behavior, and restricted behavior. Individual items are rated on a 4-point Likert scale ranging from 0 (behavior does not occur) to 3 (behavior occurs and is a severe problem). The RBS-R yields subscale scores, an overall total raw score, and a total item endorsement score. Parents were asked to complete the RBS-R during the pre and post intervention assessments. 

Children’s enjoyment and engagement were evaluated via the Physical Activity Enjoyment Scale (PACES). The PACES survey is an effective measure of children’s enjoyment across different physical activities with high reliability and internal consistency of scores [43]. 

### 2.5. Statistical Analysis of Individual Outcomes

Data for the assessment of individual outcomes (RBS-R) were analyzed in the Statistical Analysis System (SAS Version 9.4, SAS Institute Inc., Cary, NC, USA) using the linear mixed model procedure (Proc Mixed). Mixed modeling was adopted to allow for a different residual error term (i.e., a different standard deviation (SD) for the pre-post intervention change score) to be specified for boys and girls and to account for unequal variances between these two groups (i.e., difference in the effect of baseline). The overall models for subcomponents of the RBS-R compared mean changes (post minus pre) with the change score as the dependent variable and with adjustment made for baseline differences between groups.

When delivering interventions to clinical populations with a high degree of psychosocial and behavioral variability, it is also important to evaluate and identify potential individual responses to treatment alongside group-level effects. To do this, we followed the approach suggested in Hecksteden et al. [44] and formulas outlined by Hopkins [45,46]. This resulted in a two-step process.

1. To evaluate the extent of individual responses, we calculated a SD representing the net mean difference in the effect of the intervention between children. This SD representing individual responses is given by the square root of the difference between the square of the pre-post SD for the treatment group (SD_T_) and the square of the standard error of measurement (SEM) divided by the square root of two: SD_IR_ = [SDT2−(SEM2)2]. An SEM was derived from an appropriate reliability study to facilitate these calculations [47]. Uncertainty in this estimate of individual responses is evaluated by calculating its upper and lower confidence limits and an accompanying p value. The confidence limits are calculated using appropriate critical value from the t distribution, the standard error of the pre-post change score and the degrees of freedom for the SD and from the reliability study from which the SEM was derived: 2[(SDT4dfT)+((SEM2)4dfSEM)] . The *p* value is calculated from these confidence limits using the step-by-step process outlined by Altman and Bland [48].

2. To individual responders, we: (i) Adjusted the individual change scores for each child to remove the artefactual effects of regression toward the mean; and (ii) expressed the uncertainty in each child’s change score as 95% confidence limits by multiplying (SEM2) by the appropriate critical value (1.96) from the *t* distribution. The artefactual effects of regression towards the mean were removed from each child’s change scores by: (i) Mean centering baseline scores; (ii) multiplying these values for baseline by a slope representing the effect of baseline derived from statistical first principles (i.e., by  (SEM22)SD, where the denominator SD is the between-baseline SD for subjects in the control group; and (iii) adding this final effect to the child’s original change score [45,46,49]. This process results in a value for each child’s change score that is free of any contribution arising from regression towards the mean. These adjusted change scores can be illustrated on a scatter-plot with 95% confidence limits and with a highlighted area indicating trivial effects to show the proportion of positive-, negative-, and non-responders.

To evaluate the importance and uncertainty in the effects, an alpha level 0.05 was set for all analyses. Uncertainty in the estimate was therefore expressed as 95% confidence limits. The magnitudes of the effects were assessed using the following standardized scale using an updated version of Cohen’s d scales to identify substantial percentage changes over time and differences between groups: <10%, trivial; 10–30%, small; 30–50%, moderate; 50–70%, large; >70%, very large [49,50]. Following recommendations by Hopkins [46], these thresholds were halved for evaluating the size of the SDs representing individual responses.

## 3. Results

### 3.1. Study 1

To establish the feasibility of the intervention, the first study—consisting of assessments and two intervention sessions—was conducted with a small sample size (*n* = 12; 42% female). All children had prior diagnoses (provided by community providers) of ASD, and 44% were also diagnosed with Attention Deficit/Hyperactivity Disorder (ADHD). The purpose of the study was to receive qualitative feedback on the organization of the program and was focused on measuring engagement and enjoyment. Children’s enjoyment and engagement were evaluated via the Physical Activity Enjoyment Scale (PACES); 80% endorsed the statement “I enjoyed it”. In a focus group, parents described their interest in a longer intervention and their perceptions of how the intervention could be beneficial for their children, with many describing their child’s interest in and love for music as particularly relevant. Parents described many barriers to physical activity programs in the community, and indicated that having access to an intervention like Creatively Able was especially appealing. Thus, based on this positive feedback from children and parents, a short-term intervention was designed and implemented (Study 2).

### 3.2. Study 2

We had four goals for this pilot study: (1) To evaluate the feasibility of recruitment and implementation, (2) to evaluate child enjoyment of intervention sessions, (3) to evaluate target engagement (i.e., children’s self-regulation) during intervention sessions, and (4) to collect pilot data on ASD outcomes, using an ASD clinical symptom rating scale [42]. We analyzed individual responses to intervention using this rating scale to illustrate potential trends, not expecting a 4-week intervention to yield substantial or significant clinical improvements.

#### 3.2.1. Feasibility of Recruitment and Implementation

We recruited participants by distributing a flyer through a local community program serving families of children with ASD. The Study 2 sample (*n* = 8) included 4 females and 4 males with ASD, with some participants having comorbid disorders (28.5% ADHD, 14.3% Anxiety Disorders, and 14.3% a Long Term Memory Disorder). Diagnoses were reported by parents at enrollment and had been obtained through community providers as part of qualification for services at the center where recruitment was conducted. The mean participant age was 8.5 ± 2.07 years.

Parents of these eight children contacted the number on the flyer and scheduled intake appointments. Our consent and retention rates were both 100%. Participants attended eight 45-minute sessions over a four-week period. Professor Palermo conducted the sessions in a UC Irvine School of Arts dance/arts studio. Participants were evaluated before and after the four-week intervention, and sessions were video-recorded. Analysis of participation data revealed that one participant, whose symptoms were relatively severe compared to the other participants, did not engage in most group activities, although she followed along from the sidelines in some portions of the classes. This participant was nonverbal and also unable to complete certain assessment measures; thus, for some measures, only data on seven participants was available.

#### 3.2.2. Self-Reported Enjoyment

All participants completed the post-intervention PACES questionnaire (Table 1). None of the participants “Disagreed a lot” with statements that were indicative of enjoyment. Eighty-six percent of participants agreed with the statement “I enjoyed it”, and 100% of participants disagreed with the statement “I disliked it”. 

#### 3.2.3. Participant Engagement and Self-Regulation during Intervention Sessions

**Figure 1:**
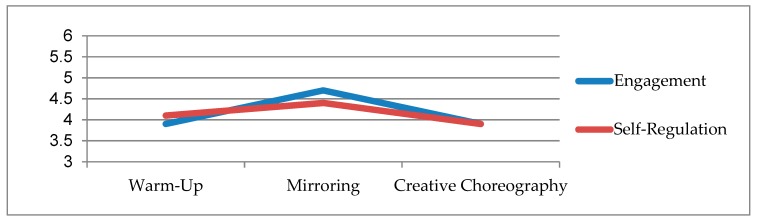
Participant engagement and self-regulation during sessions.

Ratings (Figure 1) demonstrated that during the intervention sessions, children with ASD were perceived by raters as engaged [39] and self-regulated [40,41] to a degree that would be expected for an average, typically developing child of their age (i.e., with means ranging from 3.9 to 4.7 on scales where a 4.0 represents “average” for a typically developing child). It was interesting to note that both engagement (M = 4.7) and self-regulation (M = 4.4) were highest during the mirroring activities, which as noted earlier were developed to help promote social communication by motivating children to practice key social skills such as attention, eye contact, and awareness of others.

#### 3.2.4. Improvements in Clinical Symptoms: Individual Responses to Intervention 

**Figure 2:**
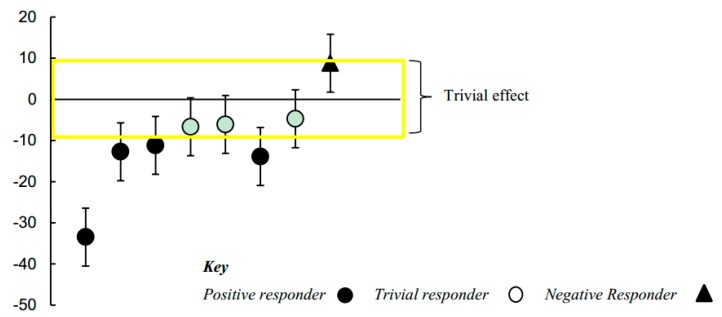
Pre-post study changes in Stereotyped Behaviors for individual children, with 95% confidence limits.

**Figure 3:**
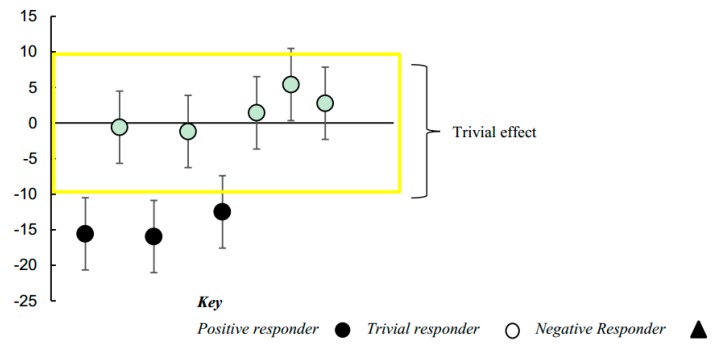
Pre-post study changes in Compulsive Behaviors for individual children, with 95% confidence limits.

The 4-week intervention yielded significant individual differences in the clinical improvements measured using the RBS-R scale. Improvements were observed across two of the six RBS-R subscales: Stereotyped (i.e., stereotypical movements) and Compulsive (i.e., lack of flexibility, adherence to strict rituals) Behaviors. There were group-level reductions in Stereotyped and Compulsive behaviors of 8% and 4%, respectively; however, interpreting the lower and upper confidence limits for these effects suggested that group-level outcomes were at most only possibly substantial (Stereotyped Behaviors: −8.13 ± 17.91; percentage change, ±95% confidence limits) (*p* = 0.20) (Compulsive Behaviors: −4.06 ± 8.50) (*p* = 0.26). Posthoc analysis revealed that there were substantial individual differences in children’s responses to the intervention. The SDs representing individual responses were both statistically significant and substantial in terms of their effect size (Stereotyped Behaviors: 12.38 ± 9.87; SD, ±95% confidence limits) (*p* = 0.02) (Compulsive Behaviors: 8.37 ± 6.93) (*p* = 0.02). These outcomes were such that we also followed up to identify and visually represent on a scatterplot the most likely individual responders with 95% confidence limits for each responder’s pre-post intervention change score. As can be seen from Figure 2, when taking into account children’s changes in Stereotyped Behaviors, there was one quite large positive responder, three small responders, three trivial responders (or non-responders), and one possible negative responder to the intervention. Changes in Compulsive Behaviors are illustrated in Figure 3. These data show that there were three clear positive responders, whilst the rest of the children exhibited trivial responses. As we expected that a brief 4-week intervention would be too short to yield large clinical improvements, these results are encouraging and point to potential areas of improvement for a longer intervention.

## 4. Discussion

### 4.1. Creatively Able: A Music and Movement Intervention for Children with ASD

This manuscript describes results from two preliminary studies of Creatively Able—a music and movement intervention for children with ASD. Our first study (Study 1) was a two-session investigation to measure both feasibility and acceptability of the intervention. Parent focus groups indicated substantial interest in participating in a longer program, and 80% of child participants agreed with the statement, “I enjoyed it”. Based on these findings, we designed and conducted Study 2. 

Participants in Study 2 completed a four-week intervention consisting of eight sessions (two per week), and data was collected on participant enjoyment, engagement, and self-regulation. Clinical data on restricted interests and repetitive behaviors (RRBs) was collected before and after participation to examine improvements in ASD symptoms. Eighty-six percent of participants agreed with statements related to enjoying the intervention (e.g., “I enjoyed it”, and “I found it fun”). No participants reported disliking the intervention. These findings are encouraging, as it suggests children enjoyed participating in the intervention. 

When child behaviors during the intervention were observed and coded, both self-regulation and engagement were rated as being similar to that expected from a typically developing child. Of particular interest, ratings of both self-regulation and engagement were numerically highest during the “mirroring” activity, in which children were instructed to copy the actions and movements of a partner. Given that social-communication deficits are one of two primary symptoms of ASD, one might expect that engagement and self-regulation would be low during a task that requires children to directly engage with a partner and mirror their movements. It is possible that children with ASD have less difficulty with tasks involving social engagement when music is involved, though our study was not designed to explicitly address this question. Future studies should consider directly measuring child behaviors during a mirroring task that either involves music or is done in the absence of music. 

Finally, we measured restricted and repetitive behaviors (RRBs) before and after participation in the intervention. The presence of RRBs is the second primary symptom of ASD, and improvements in these behaviors would represent a clinically meaningful treatment effect. Of six subscales (Stereotyped behavior, Self-injurious behavior, Compulsive behavior, Routine behavior, Sameness behavior, and Restricted behavior), significant symptom reduction was found in both the Stereotyped and Compulsive behavior scales. The Stereotyped behavior subscale measures repetitive motor movements often associated with ASD (e.g., head, finger, and body movements, and stereotyped use of objects). The Compulsive behavior subscale measures behaviors often associated with obsessive–compulsive disorder, but which are also observed in ASD (e.g., compulsive checking, counting, washing, or ordering). Individual responses to intervention were measured, and we found that this change was largely driven by four positive responders for the Stereotyped behavior subscale, and three positive responders for the Compulsive behavior subscale. Measuring individual treatment response is crucial for understanding how and why certain children respond to specific interventions while others do not. Future studies with Creatively Able and other music or movement interventions should explore factors that can help predict who will be a positive responder for a particular intervention target (e.g., factors observable at baseline, symptom characteristics). It is important to note that with a sample size of 8 and a time-limited intervention (four weeks), we did not expect to observe clinically meaningful improvements in RRBs. The fact that change was observable after a short time in such a small sample is encouraging for future work with a larger sample and longer duration intervention. 

### 4.2. Studying Qualitative or Contextual Features of Physically Active Interventions

Our intervention suggested that Creatively Able, a physically active intervention, may be beneficial for self-regulation for children with ASD, a finding that is consistent with the literature for typically developing children [51]. The majority of studies examining the effects of PA interventions on executive function or other cognitive outcomes have focused on the effectiveness of aerobic exercises with or without additional cognitive challenges, without elucidating on the qualitative characteristics of their program or the contextual factors related to the program [19]. An important characteristic of our intervention is that it combined music with rhythmic movement patterns, as well as promoted working with partners through social activities, such as the mirroring activity. The use of music and positive social interactions as integral characteristics of PA programs have been found to be associated with positive affect and enjoyment in children [52] and have been identified as key constructs when planning or promoting quality PA sessions in youth [53]. Deriving satisfaction from a PA program and experiencing positive affect has been proposed to be associated with higher levels of engagement and self-regulatory skills [20,54,55]. 

It is also important to emphasize that since the intervention program had multiple qualitative characteristics, it was not possible to parse out which component was most influential for children with ASD. The need to further study qualitative or contextual components of physically active interventions that are understudied, such as those involving music, rhythm, and social interactions, has been documented in the literature [19,20] and this pilot intervention is a positive step towards that direction. However, more research in this area is warranted. Thus, as a next step, we are preparing to conduct a randomized intervention study, by providing Creatively Able over a longer term (ten weeks) to further study improvements in ASD symptoms as well as potential neural and behavioral mechanisms underlying changes.

### 4.3. Studying Individual Responses to Intervention

Despite shared core symptoms, there is significant variability in behavioral presentation, or phenotype, of children with ASD. Due, in part, to this variability, it has been difficult for researchers to identify specific interventions as being effective for all children with ASD. While there are a number of evidence-based interventions for children with ASD, not all children benefit equally from all interventions [56,57]. This causes difficulties for families trying to decide which interventions to try, as behavioral interventions are often time consuming and costly, and there is no clear way to predict whether one’s child will benefit from a specific intervention prior to trying it. In a large-scale review of available interventions for children 0–12 with ASD, Weitlauf et al. [57] noted that a critical avenue for future research is the ability to better predict whether children will respond to specific interventions. We demonstrated how researchers can examine individual responses to intervention using the framework outlined in Hecksteden et al. [44] and formulas outlined in Hopkins [45,46]. Analysis of individual responses provided: (1) A standard deviation which shows the amount by which effects differ between children; and (2) the proportion of children in the treatment group who were clear positive responders. Both of these outcomes are critical to report when delivering interventions to clinical populations with a high degree of psychosocial and behavioral variability. Examination of individual responses to intervention can help investigators further refine intervention protocols to meet the needs of potential non-responders and also potentially identify mechanisms of change that could be further studied to inform scientific knowledge about how interventions affect various domains of functioning. 

## 5. Conclusions

Our research supports the feasibility of recruitment and implementation of Creatively Able and documents interest in participating among children with ASD and their families. Results from both studies indicated that children with ASD enjoyed Creatively Able and were engaged during intervention sessions; moreover, results suggested target engagement (e.g., self-regulation), which we predict will lead to improvements in self-regulation and outcomes associated with self-regulation. Analyses of group and individual outcomes showed a trend toward improvement in ASD symptoms (i.e., a reduction in repetitive behaviors including stereotyped behaviors and compulsive behaviors, such as lack of flexibility), which are related to self-regulation. We illustrated how we can move beyond examining outcomes only at the group level and can begin to better understand individual responses to interventions. Finally, our research has implications for further examination of contextual features of different physical activity interventions, such as the inclusion of music as well as activities designed for specific populations. 

## Figures and Tables

**Table 1:** Participant responses on the Physical Activity Enjoyment Scale (PACES) questionnaire.

Item	Disagree a Lot (%)	Disagree (%)	I Am Not Sure (%)	Agree (%)	Agree a Lot (%)
**I enjoyed it**	0	0	14	29	57
**I found it fun**	0	14	0	29	57
**It was very pleasant**	0	14	29	29	29
**It gave me energy**	0	29	0	43	29
**My body feels good**	0	14	14	14	57
**It feels good**	0	0	0	43	57
**I got something out of it**	0	14	14	29	43
**It was very exciting**	0	14	0	14	71
**It gave me a strong feeling of success**	0	29	14	14	43
**I feel bored**	43	14	14	0	29
**I disliked it**	71	29	0	0	0
**It made me sad**	57	14	14	14	0
**It was not fun at all**	71	0	14	14	0
**It frustrated me**	71	0	0	29	0
**It was not at all interesting**	43	14	29	0	14
**I feel as though I would rather be doing something else**	43	14	29	14	0

Note: Due to rounding, not all lines will total 100%.
